# Polyaniline Nanofibers-Embedded Gold Nanoparticles Obtained by Template-Free Procedure with Immobilization Prospects

**DOI:** 10.3390/s21248470

**Published:** 2021-12-18

**Authors:** Joaquín Rafael Crespo-Rosa, Alfonso Sierra-Padilla, Juan José García-Guzmán, David López-Iglesias, Dolores Bellido-Milla, José María Palacios-Santander, Laura Cubillana-Aguilera

**Affiliations:** 1 Department of Analytical Chemistry, Institute of Research on Electron Microscopy and Materials (IMEYMAT), Faculty of Sciences, Campus de Excelencia Internacional del Mar (CEIMAR), University of Cadiz, Campus Universitario de Puerto Real, Polígono del Río San Pedro S/N, 11510 Puerto Real, Cádiz, Spain; joakincrespo@hotmail.com (J.R.C.-R.); alfonso.sierra@uca.es (A.S.-P.); david.lopeziglesias93@gmail.com (D.L.-I.); dolores.milla@uca.es (D.B.-M.); laura.cubillana@uca.es (L.C.-A.); 2 Instituto de Investigación e Innovación Biomédica de Cadiz (INiBICA), Hospital Universitario ‘Puerta del Mar’, Universidad de Cadiz, 11009 Cádiz, Spain

**Keywords:** conducting polymers, polyaniline nanofibers, conducting polymers-gold nanoparticles nanocomposite, high-energy ultrasound, Sonogel-Carbon, electrochemical biosensors

## Abstract

In this work, template-free nanostructured conducting polymers (nCPs)-embedded gold nanoparticles (AuNPs) from aniline, thiophene and 3,4-ethylenedioxythiophene have been prepared via a one-pot sonochemical method. The synthesis of the nanocomposite (nCPs-AuNPs) was achieved in a short period of time (5–10 min), by applying high-energy ultrasound to an aqueous mixture of a CP precursor monomer and KAuCl_4_, in the presence of LiClO_4_ as dopant. The synthesis process is simpler, greener and faster in comparison to other procedures reported in the literature. Remarkably, bulk quantities of doped polyaniline PANI-AuNPs nanofibers were obtained. Subsequently, they were characterized by scanning electron microscopy (SEM), transmission electron microscopy (TEM), energy-dispersive X-ray spectroscopy (EDS) and Fourier transform infrared spectroscopy (FTIR), as well as by cyclic voltammetry (CV) and electrochemical impedance spectroscopy (EIS). PANI-AuNPs nanofibers were also employed as immobilization matrix for a benchmark enzyme, glucose oxidase (GOX). Finally, glucose was determined in real samples of white and red wines by using the so-obtained GOX-PANI-AuNPs/Sonogel-Carbon biosensor, providing outstanding recoveries (99.54%). This work may offer important insights into the synthesis of nanostructured conducting polymers and also stimulates the exploration of the applications of these nanocomposites, especially in research fields such as (bio)sensors, catalysis and composite materials.

## 1. Introduction

The use of conducting polymers represents one of the most important pathways for the preparation of modified electrodes to produce sensors, biosensors or energy storage systems. Their fast development is due to the successful combination of interesting mechanical features such as high flexibility and low density, with excellent electrical properties, similar to metal conductivity. Their synthesis can be achieved by different methods: direct synthesis [1], electrochemical synthesis [2], monomers chemical oxidation [3] or by other means, such as plasma [4] or electrospinning [5]. Conducting polymers possess the conjugated double-bonded backbone that extends electronic orbitals on the whole structure, providing an excellent electronic conductivity after suitable doping [6]. In fact, one of the most notable properties of intrinsically conducting polymers, such as polypyrrole, polyaniline (PANI), polythiophene (PT) and relevant derivatives, is that their use as modifiers of electrode surfaces makes them capable of activating electrocatalytic redox processes [7].

Alternatively, the additional use of catalytically active substances during their synthesis produces an improvement in the electron transfer process [8]. Particularly, in the case of conducting polymers modified with metal nanoparticles (e.g., Au or Pt), it has been demonstrated that there is a synergy between the two materials whose composite improves the electroanalytical characteristics and applications [9]. On one hand, these CPs work as simple templates for the incorporation of the secondary component; on the other hand, their physical characteristics together with electrostatic interactions can influence the electrocatalytic reaction [10]. In the literature, there are numerous examples of their use in electroanalysis: a poly(3,4-ethylenedioxythiophene):polystyrene sulfonate-gold nanoparticles (PE-DOT:PSS-AuNPs)-based sensor for hydrogen sulphide determination [11]; Au@PANI and Au@polypyrrole (PPy)-based sensor for As(III) detection [12]; a SeS_2_ incorporated Co met-al-organic framework (MOF) and Au@PANI nanocomposite used to build a molecularly imprinted polymer (MIP) to determine patulin mycotoxin [13]; a AuNPs-PANI-based sensor for dopamine detection in presence of ascorbic acid and uric acid [14]; a AuNPs-PEDOT nanocomposite-based sensor for studying electrocatalytic properties in glucose oxidation in alkaline media [15]; a review about recent advances in electrochemical sensors and biosensors based on conducting polymers (PEDOT, PANI, PPy, poly-indole, etc.) doped with different nanomaterials including carbon nanomaterials, metal/metal oxide nanoparticles and quantum dots [16]; and the determination of dopamine and cathecol with a multiwalled carbon nanotube-doped conducting polymer-tyrosinase biosensor [17].

Nanostructured conducting polymers (nCPs) are understood as polymers which, besides presenting electrical conductivity, show a homogeneous structure at the nanoscale. Due to their high surface area to volume ratio, they present a wide variety of unique features, such as enhanced optical, magnetic, thermal, and electrical properties. nCPs can be in the shape of nanotubes, nanowires or nanoparticles [1] and combine the electronic properties of inorganic semiconductors and metals with the good processability and tunable surface functionalities of polymers, which confers on them an impressive potential for multiple applications, such as drug delivery, surface coatings, nanoreactors, catalysis, filtration and electrochemistry [18]. Particularly, these special characteristics have made them suitable as base materials for the manufacture of electrochemical sensors, as extensively reported in the review about the application of CP nanostructures in electrochemical biosensors [19] and in the work of Popov et al. [20] about the development of an amperometric glucose biosensor using a nanocomposite based on reduced graphene oxide and PANI nanofibers. This kind of analytical device offers elegant routes for interfacing at the molecular level, chemical or biological recognition events and electronic signal-transduction processes, as reported in these two recent reviews about nanomaterials and conducting polymer-based (nano)composites for electrochemical and E-tongues/noses applications [21,22].

Furthermore, nCPs can be easily functionalized, since they exhibit high conjugation of the polymer chains, causing a “doping/de-doping” process. This results in a substantial increase in conductivity, as well as an increase in its specific area, which plays an important role in the sensitivity of the developed device. Due to this possibility of functionalization, nCPs are frequently used in the form of nanocomposites, in combination with other nanomaterials such as metal nanoparticles. So far, the main routes of synthesis of nCPs modified with metal nanoparticles consist of methods involving several intermediate steps: electrospinning [5,23], the use of templates for the direct formation of nanofibers [24,25], chemical methods [26], and electrochemical methods [27].

Currently, the application of focalized ultrasound irradiation is an alternative, very attractive tool. Its main advantages in the polymerization process are the absence of external chemical initiators and the possibility of bulk polymerization [28]. In general, the chemical consequences of ultrasound irradiation arise from acoustic cavitation, which provides the primary mechanism for sonochemical effects. In brief, cavitation serves as a means to concentrate the diffuse energy of sound into an exceptional set of conditions under which the one-step formation of nCPs-AuNPs is promoted and favored [29]. Ultrasonic methods are usually based on ultrasound baths [30], but this approach possesses several flaws. For example, in general, the reaction takes a long time (several hours or even more) with intermediate steps (which increases complexity), high energy requirements are also needed. Hence, the processes are not environmentally friendly (incompatible with Green Chemistry and the Sustainable Development Goals within 2030 Agenda) and, in some cases, it needs the use of accelerators such as: chemical compounds, macromolecules, UV or γ radiation [31,32,33], respectively. In order to solve these disadvantages, high-energy ultrasound probes are rising as a more efficient and green alternative.

In this paper, the possibility of finding a fast, green, one-pot, simple and template-free synthesis method to obtain nCPs modified with metal nanoparticles is studied. The synthesis procedure is accomplished in a single step (one-pot and simple) by applying a very reduced period (fast) of high-energy ultrasound (green) to an aqueous mixture of the monomer and the metal salt in the presence of a dopant (template-free). This method is based on the direct electron exchange between metal salt as acceptor, and monomers as donor. To our knowledge, there is only one application of high energy ultrasound for the one-pot synthesis of nCPs-AuNPs nanocomposite, specifically PANI and Au-PANI, obtained by Gedanken and Sivakumar in 2005 [33]. However, the synthesis process employs H_2_O_2_ as accelerator and takes several hours (at least 3 h) instead of 5-10 min, hence requiring much more energy than our proposal. The structural characterization of the nCPs-AuNPs nanocomposite obtained following the above-mentioned synthetic process is also reported here by using different instrumental techniques Finally, PANI-AuNPs nanofibers were employed as immobilization matrix for a benchmark enzyme, glucose oxidase (GOX) in order to determine glucose in wine samples with excellent recoveries.

## 2. Materials and Methods

### 2.1. Reagents and Materials

The following chemicals were purchased from Sigma–Aldrich/Merck (Darmstad, Germany): Potassium chloride >99% (KCl; 7447-40-7), potassium tetrachloroaurate > 99.995% (KAuCl_4_; 13682-61-6), lithium perchlorate >99.99% (LiClO_4_; 7791-03-9), aniline > 99.5% (62-53-3), commercial polyaniline (emeraldine base) (25233-30-1), thiophene > 99% (110-02-1), 3,4-ethylenedioxythiophene > 97% (EDOT; 126213-50-1), sodium dodecyl sulfate ˃99.5% (SDS; 151-21-3), *L*-ascorbic acid > 99.0% (AA; 50-81-7), glucose oxidase from *Aspergillus niger* (158.9 U/mg; 9001-37-0) and methyltrimethoxysilane > 98% (MTMOS; 1185-55-3). HCl 37% and HClO_4_ 70% were from Panreac (Barcelona, Spain). D(+)-glucose monohydrate > 99.0% (14431-43-7), potassium dihydrogen phosphate (KH_2_PO_4_; 7778-77-0) and dibasic potassium phosphate (K_2_HPO_4_; 7758-11-4) for phosphate-buffered solution (PBS), potassium hexacyanoferrate(II) trihydrate (K_4_Fe(CN)_6_·3H_2_O; 14459-95-1) and potassium hexacyanoferrate(III) (K_3_Fe(CN)_6_; 13746-66-2) were from Fluka (Buchs, Switzerland). Graphite powder natural, high purity -200 mesh (dimension < 74 µm), 99.9999% (metal basis; 231-955-3), was from Alfa-Aesar (Johnson Matthey GmbH, Germany). All the reagents were used as received without further purification. Nanopure water was obtained through a Milli-Q system (18 MΩ cm, Millipore, Bedford, MA, USA). Glass capillary tubes, i.d. 1.15 mm, were used as the bodies of the composite electrodes. The nitrogen used for getting inert atmospheres and deaerating solutions in the electrochemical measuring cell was N-55 type.

### 2.2. Instrumentation

The synthesis of the nanostructured conducting polymer material, as well as the ultrasonic synthesis of Sonogel-Carbon (SNGC) material used as electrode transducer for the electrochemical measurements, were carried out by irradiating with a high energy ultrasound generator, SONICATOR 4000, from Misonix (Misonix, Inc., Farmingdale, NY, USA), equipped with a 13 mm titanium tip that provides a maximum power of 600 W (20 kHz).

Centrifugation of the obtained nanocomposites was performed in a Biocen 22 R centrifuge, from Ortoalresa-Álvarez Redondo, S.A (Daganzo de Arriba, Madrid, Spain).

UV–visible measurements were made using a Jasco V-550 (Easton, MD, USA) UV-visible spectrophotometer. For this purpose, samples from the liquid phase of the nCPs/AuNPs nanocomposite suspensions were measured in the range of 300–700 nm.

Fourier transform infrared spectroscopy was carried out using a Shimadzu FTIR-8400S spectrophotometer (Shimadzu, Kyoto, Japan) with a resolution of 4 cm^−1^ in the region from 2000 to 600 cm^−1^.

Scanning electron microscopy (SEM) of the materials surface was performed on a Quanta200 (FEI Company, Hillsboro, OR, USA), normally operating at 20 kV and equipped with an INCA 350 system (Oxford Instruments, Abingdon, UK) to perform X-ray energy dispersive spectroscopy (EDS).

Transmission electron microscopy (TEM) studies were carried out on a JEOL JEM-2010F (Jeol, Tokyo, Japan) microscope, equipped with a field emission gun, a scanning-transmission electron (STEM) module, a high angle annular dark field detector (HAADF) and an X-ray energy dispersive spectroscopy (EDS) microanalyzer. The microscope was operated at 200 kV and in the STEM mode a 0.5 nm probe was used.

SEM characterization was accomplished directly onto the electrode surface. For this purpose, the filled tip of glass capillary tubes (ca. 8–10 mm length) of the SNGC electrodes previously drop-casted with the corresponding volume of nCPs-AuNPs nanocomposite colloidal solutions were cut and inserted in SEM sample holders with a hole previously drilled on its surface to host the capillary tubes. Concerning TEM and HAADF-STEM characterization, an appropriate volume of the colloidal solution of every nCPs-AuNPs nanocomposites was drop-casted onto copper-carbon grids employed as sample holders. The as-prepared sets of samples were then put into the corresponding electron microscope chamber for characterization.

The voltammetric measurements were made on a potentiostat/galvanostat Autolab^®^ PGSTAT30 (Ecochemie, Utrecht, The Netherlands) connected to a personal computer and a 663 Metrohm VA Stand module, using the software GPES (General Purpose Electrochemical System) 4.9 ver. for waveform generation, data acquisition and elaboration. The experiments were carried out in a single-compartment three-electrode cell, at room temperature (25 ± 1 °C). The counter electrode was a platinum wire, and a silver/silver chloride/3M KCl electrode was used as the reference electrode. The SNGC composite-filled glass capillary tubes, conveniently deposited with the adequate recognition element (bare electrode, nCPs-AuNPs, enzyme-nCPs-AuNPs, etc.), were used as the working electrode. Finally, this equipment also has an integrated FRA module to perform electrochemical impedance spectroscopy (EIS) measurements: frequency range 0.1–10 kHz using a sinusoidal excitation signal (single sine) with excitation amplitude (ΔE_ac_) of 5 mV.

### 2.3. Synthesis of the Conducting Polymer Nanofibers/Gold Nanoparticles (nCPs-AuNPs) Nanocomposite

The synthesis of the conducting polymer nanofibers/gold nanoparticles nanocomposite (nCPs-AuNPs) is a fast, green, one-pot and simple procedure based on the application of high energy ultrasound to an aqueous mixture of the monomer and the metal salt in the presence of a dopant. The synthetic route is described in the next sequence. In a glass vial, containing 5 mL of 50 mM LiClO_4_ (aqueous solution) used as dopant, certain volumes of the following reagents were added: (i) an adequate amount of the precursor metal salt (KAuCl_4_) with concentrations ranging from 0.1 to 1 mM (optimal value = 0.5 mM); and (ii) 12 µL, 12 µL or 4 µL of the commercial solution of the precursor monomer: aniline, thiophene and 3,4-ethylendioxythiophene, respectively, giving the following optimized concentration values for each monomer: 26 mM, 30 mM and 7.5 mM, respectively. The monomer mixture was then irradiated with ultrasound for 5 min at a maximum output power of 32 watts. The final product, a heterogeneous dispersion of the nanocomposite (nCPs-AuNPs), is composed of a water soluble colloid and a solid precipitate, the precipitate richer being in gold nanoparticles, according to [25]. Precipitation of the nanostructure occurs as the polymer chain length increases and the solubility limit of the polymer/metal composite is exceeded. Prior to being used in sensing devices, nCPs-AuNPs nanostructures are centrifuged at 10,000 rpm for 5 min to remove the excess of conducting polymers precursors. The precipitate obtained was then re-dispersed in 5 mL of Milli-Q water for PEDOT- and PT-AuNPs and in 5 mL of Milli-Q water acidified with HClO_4_ at pH 3 for PANI-AuNPs.

### 2.4. Preparation Procedure of the Sonogel-Carbon Electrodes

To electrochemically test the materials, Sonogel-Carbon electrodes were used as transducers due to their biocompatibility and versatility for building electrochemical (bio)sensors [34]. To prepare the SNGC electrodes, the procedure described in the literature was used [35]. Briefly, a mixture of 500 μL of methyltrimethoxysilane (MTMOS) and 100 μL of 0.2 M HCl was irradiated with ultrasound for 10 s. Then, 0.5 g of graphite powder was added to the obtained sonosol, and adequately dispersed. After several minutes, the resulting material acquires enough consistency to fill the glass capillary tubes. After 24 h, the surface of the electrodes could be polished, before modification, with fine grained sandpaper (waterproof silicon carbide paper) No. 1200 (Struers, Madrid, Spain) to remove extra composite material, wiped gently with weighing paper, thoroughly washed with Milli-Q water, and allowed to dry at room temperature. Finally, a copper wire was inserted as the electrical contact into the electrodes, and the electrodes were ready to use. As mentioned in Section 2.1, the glass capillary tubes used as the bodies of the composite electrodes possesses an internal diameter of 1.15 mm; hence, the geometrical area is 1.04 × 10^−2^ cm^2^.

### 2.5. Electrochemical Pre-Treatment of Sonogel-Carbon Electrodes

Prior to the deposition of the nanocomposite materials on the surface of the SNGC electrodes, they were electrochemically pre-treated by dipping them into 0.05 M sulphuric acid solution in the electrochemical cell. The SNGC electrodes operating as working electrodes were polarized in CV from −0.5 to +1.5 V for 5 scans at a scan rate of 0.05 V·s^−1^. Electrodes with similar current backgrounds were selected, carefully washed with Milli-Q water and dried at room temperature.

### 2.6. Modification of the Sonogel-Carbon Electrodes with the Nanocomposite Materials and Characterization by the Different Instrumental Techniques

The modification of the SNGC electrodes was accomplished by depositing different aliquots of the nCPs-AuNPs solution (2, 6 or 10 µL) onto the electrode surface. After drying, the nCPs-AuNPs-SNGC modified electrodes were used for electrochemical measures. Prior to the drop-casting process onto the SNGC surface, the re-dispersed colloidal solution was gently irradiated in an ultrasound bath for 30 s and then, the corresponding volume was pipetted from the vial.

For characterization purposes, 50 µL of the nCPs-AuNPs solution were deposited over a glass and allowed to dry at room temperature. Then, characterization by the corresponding instrumental technique was performed.

### 2.7. Fabrication of the GOX-PANI-AuNPs Biosensor

The GOX-PANI-AuNPs nanocomposite-based biosensor was prepared by dissolving the appropriate amount of GOX in 10 µL of PBS 0.1 M (pH 6.8). Then, the enzyme solution was mixed with 10 µL of PANI-AuNPs suspension. Once homogenized, 2 µL of GOX-PANI-AuNPs was deposited onto the surface of a SNGC electrode. The biosensor was dried at room temperature. When not in use, the biosensor was stored in buffer solution at 4 °C.

### 2.8. Electrochemical Assessment of the GOX-PANI-AuNPs Biosensor

Capacity and mechanism studies were performed by using cyclic voltammetry, carried out in different media: 0.1 M phosphate-buffered solution (PBS) pH 7.2 in a 0.1 M KCl solution and 5 mM of K_4_Fe(CN)_6_ in a 500 mM KNO_3_ solution, respectively. Impedance assays were performed as well. The impedance characterization was achieved using an aqueous solution (pH 5) containing 5 mM K_4_Fe(CN)_6_/K_3_Fe(CN)_6_, and 500 mM KNO_3_, in the frequency range from 10 kHz to 0.05 Hz, with 5 mV amplitude of the sine wave, at a bias potential of 0.22 V (the potential of the redox couple).

### 2.9. Determination of Glucose in Wines by Using the GOX-PANI-AuNPs Biosensor

Finally, red and white wines were collected from a local winemaker to be used as real samples. Each wine possessed a different glucose concentration value: 7.48 g/L and 4.55 g/L, for red and white wines, respectively. They were determined by HPLC and taken as reference values for comparison purposes, when using the nCPs-AuNPs nanocomposite material and electroanalytical techniques. For the glucose determination, the wine samples were properly diluted and added to the electrochemical cell. Then, signals were recorded by using cyclic voltammetry in the range −0.1 to −0.6 V at 100 mV/s.

Statistical analysis in terms of relative standard deviation (RSD) of the spiked and real samples tested with the biosensor device were performed considering the average value of three replicated measures, unless different specifications are properly given in the text. Excel^®^ software was employed for this purpose. Other parameter calculations (such as limit of detection and quantitation) are detailed in the corresponding section.

## 3. Results and Discussion

### 3.1. Optimization of the Parameters of the Sonochemical Synthesis

The proposed sonosynthesis of the nCPs-AuNPs is based on the direct electron transfer between the metal salt and the precursor monomer, leading to a nanostructured conducting polymer. Metal ions solved in the aqueous solution, in the presence of LiClO_4_ as doping substance, work as electron acceptors, which have been donated by the monomers employed in each case. The metal salt used in all syntheses was KAuCl_4_.

The first material developed in this work by following the high energy ultrasound-assisted procedure consists of polyaniline containing AuNPs (PANI-AuNPs) and, subsequently, the synthesis is extended to other nanostructured conducting polymers, such as polythiophene (PT-AuNPs) and poly(3,4-ethylenedioxythiophene) (PEDOT-AuNPs). After synthesis, the PANI-AuNPs dispersion obtained shows two phases: a dispersion of nanofibers in the aqueous medium and a precipitate, also possessing fiber-shaped morphology, composed of heavy chains. Huang et al. [36] suggest that the formation of these nanofibers occurs primarily in the initial stages of the formation and growth of the nanoparticles; later, nanoparticles grow inside the polymer, being trapped and stabilized there, which is in consonance with literature [37].

The optimized synthesis parameters were: the kind of dopant, concentration of the metal salt and the precursor monomer, and time and energy applied by the ultrasound generator.

#### 3.1.1. Dopant Choice

According to the literature, dopant species are necessary for synthesis, since they act as electron carriers during the sonochemical synthesis [38]. Thus, the doping process favors the generation of electronic levels in the forbidden zone of the band structure of the synthesized polymer, improving its electrical conductivity, since the synthesis of emeraldine form is promoted. In this work, several assays were carried out using LiClO_4_ and sodium dodecyl sulfate (SDS) as dopants. In the case of SDS (5 mM), the results were not expected: the most important fraction of PANI was not in the emeraldine form, according to UV-vis spectra (not shown), resulting in a less conductive nanocomposite; moreover, STEM micrograph (not shown) showed a less ordered structure for the final material. According to literature, as SDS is an anionic surfactant, it increases the surface tension, which leads to a decrease in the uniformity of the nanostructure and leads to shorter and more interconnected PANI-AuNPs nanofibers. This interconnection increases the polarons and bipolarons density on the nanofibers; hence, conductivity of the nanomaterial obtained is negatively affected [39,40].

**Figure 1:**
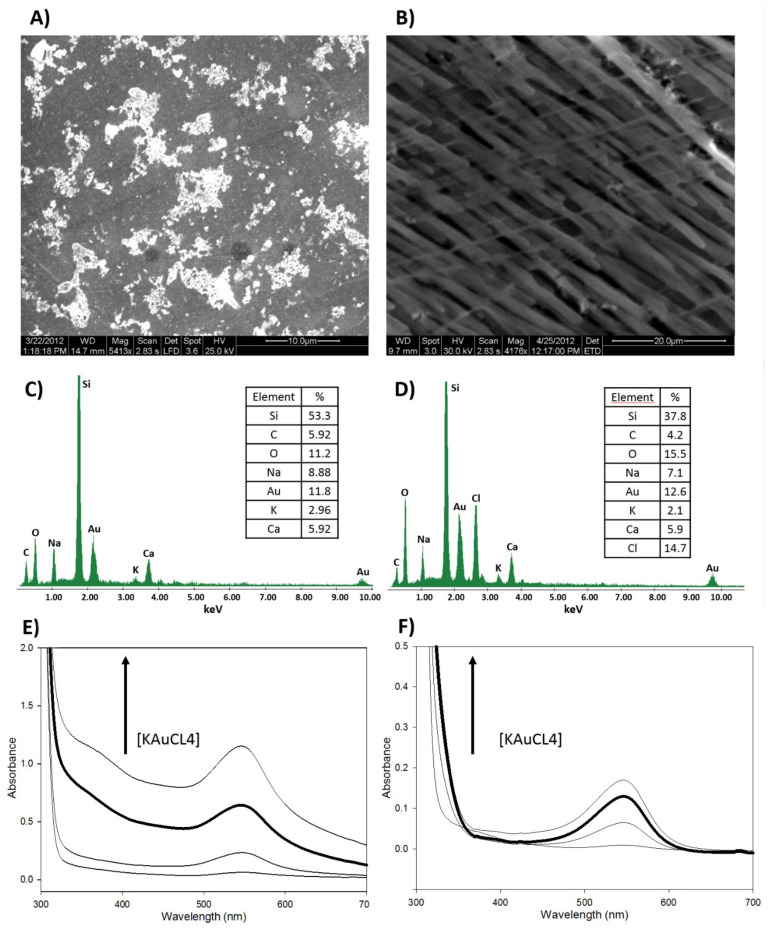
Comparison of the PANI-AuNPs nanocomposites obtained without dopant (**left**) and using LiClO_4_ 0.05 M as dopant (**right**). SEM micrographs: (**A**) without dopant; (**B**) using LiClO_4_ 0.05 M as dopant. EDS analysis of PANI-AuNPs nanocomposite obtained: (**C**) without dopant; (**D**) using LiClO_4_ 0.05 M as dopant. UV-Vis spectra of PANI-AuNPs nanocomposite obtained: (**E**) without dopant; (**F**) using LiClO_4_ 0.05 M as dopant, at different concentration values of the metal salt (0.1, 0.25, 0.5 and 1 mM KAuCl_4_).

The use of LiClO_4_ as dopant mostly leads to the emeraldine conductive form, as demonstrated elsewhere [41,42] by EDS analysis: the amount of oxygen present in the structure is higher when the synthesis is carried out using some of the most common dopants. In this work, different concentration values of LiClO_4_ were tested, having been added to the initial reaction mixture. The concentration value that offered the best results was 0.05 M. Figure 1 shows the SEM images and EDS analysis of PANI-AuNPs nanofibers obtained by sonochemical synthesis. Only in the case where the dopant was used (Figure 1B) were the PANI-AuNPs nanofibers obtained. In this case, the nanocomposite is composed of well-defined conducting polymer nanofibers, where the metal nanoparticles are uniformly distributed throughout the nanostructure. In the absence of LiClO_4_ as dopant (Figure 1A), a non-defined PANI structure was obtained spotted by AuNPs clusters of different sizes. In Figure 1C,D, the Si, O and C peaks correspond mainly to the silicon oxide network composing the Sonogel-Carbon material, with C as a massive modifier. PANI presence also contributes to C and O peaks. The higher content of oxygen observed in the EDS analysis for the synthesis with dopant also may corroborate an important fraction of PANI in the emeraldine form [43], as compared in the tables of elemental composition. In addition, K and Au peaks come from the AuNPs precursor, hence demonstrating the presence of AuNPs in both cases, especially in the case of nanofibers (slightly higher Au percentage). The majority of the Cl percentage is due to the presence of the dopant (LiClO_4_) involved in the synthesis process of the CP; that is why Figure 1C (synthesis without dopant) does not show this element. Ca and Na supposedly have an environmental origin, probably during sample preparation or during the measuring process.

#### 3.1.2. Metal Salt and Monomer Concentration

The amount of metal salt used in the sonochemical synthesis is crucial since it leads to the formation of the nanofibers and, at the same time, it determines the size of the metal nanoparticles formed as well [44].

To determine the reaction stoichiometry of the sonochemical synthesis, a series of KAuCl_4_ solutions were prepared in 5 mL shell vials: 0.1, 0.25, 0.5 and 1 mM. The concentrations of the precursor monomer were optimized in 26 mM, 30 mM and 7.5 mM for aniline, thiophene and 3,4-ethylenedioxythiophene, respectively, for each nCPs-AuNPs nanocomposite synthesized. These quantities implied to start the synthesis with the maximum amount of monomer that generated the lowest amount of nanocomposite precipitated or, in other words, the more stable suspension of the nanocomposites. In Figure 1E,F, different UV-Vis corresponding to the synthesis of PANI-AuNPs nanofibers, with (Figure 1F), without dopant (Figure 1E) and varying the concentration of the metal salt are shown, as an example. In both cases, the surface plasmon resonance (SPR) band characteristic of the AuNPs appears at 550 nm. The UV-Vis spectra obtained when using LiClO_4_ as dopant in the sonosynthesis shows better-defined bands, with almost completely planar baselines. Moreover, the SPR bands are less intense here, which can be attributed to the absence of AuNPs in solution when it is compared with Figure 1E, in which AuNPs are mainly in colloidal form. In this last case, when characterizing the deposit of the material with SEM, the agglomeration of AuNPs on the surface of a non-defined PANI structure is evident (as seen in Figure 1A). In case of the doped PANI-AuNPs nanofibers (Figure 1B), an important fraction of AuNPs is preferably located on the surface or embedded in the structure of the nanocomposite, as it will be shown later with TEM characterization.

Figure 1F is also focused on the optimization of the concentration value of the metal salt to obtain the PANI-AuNPs nanocomposite, chosen as an example. For each concentration value, the diameter of the nanofibers of the nanocomposite obtained was measured and characterized. The concentration value that offered a compromise between stability and structure was 0.5 mM of KAuCl_4_ (bold line). At this value, the AuNPs offered a more stable SPR band at 550 nm, and the average diameter of the nanofibers was less than 100 nm. In the Structural Characterization section, it will be demonstrated that the diameter of the PANI nanofibers can be controlled by changing the concentration of aniline, with lower concentrations producing fibers with thinner diameters. The results of the UV-Vis studies may also give evidence of the presence of zerovalent metal particles in the fibers generated indicating that the polymerization reaction involves reduction of gold as well. This appears to be consistent with other papers of gold-oxidized conducting polymer preparations where a product of gold reduction is incorporated into a conducting polymer, as reported in [45].

#### 3.1.3. Ultrasound Irradiation Time and Energy Applied to the Mixture

The effect of time and high energy ultrasound on the formation of nCPs-AuNPs was also investigated. The study was carried out by varying the output power of the ultrasonic probe from 10 to 80% of its maximum power. The time of ultrasound application was also evaluated in a time interval from 1 to 10 min. Time and power values higher than 5 min and 40% of amplitude, respectively, provided and excessive amount of energy and degrade the polymer nanofibers obtained. Values lower than 5 min of sonication time and 30% of amplitude did not reach the successful formation of the nanocomposite. Hence, 5 min and 40% of amplitude were chosen as optimal synthesis parameters for further studies. After synthesis, the resulting mixture was characterized by means of UV-Vis spectroscopy. In Figure 1E,F, the presence of the SPR band centered at 550 nm in the spectra confirms the formation of AuNPs [44]. Thus, the maximum power applied to reach the maximum SPR absorbance value was optimized at 32 W. This amount of energy is applied to 5 mL of 0.5 mM KAuCl_4_, 50 mM LiClO_4_ and the corresponding precursor monomer amount, in each case, placed in a glass vial. Hence, the amount of energy and time of synthesis was similar for the three nCPs-AuNPs.

#### 3.1.4. Time Stability of the nCPs-AuNPs Nanocomposites

Finally, a study of the stability of the developed nanofibers was carried out by periodically recording the UV-Vis spectra of the suspensions obtained under optimized conditions. When not in use, they were stored at room temperature and in darkness. In all cases, no variation of the absorption band can be observed up to twenty days after the synthesis. Specifically, in case of PANI-AuNPs colloids, monitoring of the UV/vis spectrum of the solution was carried out after 1, 4, 13 and 20 days after the synthesis, obtaining no significant differences in them, which indicates good stability in time of the colloidal solution corresponding to PANI-AuNPs used in this work. Accordingly, the nCPs-AuNPs nanocomposites showed good stability.

### 3.2. Structural Characterization of the nCPs-AuNPs Nanocomposites

**Figure 2:**
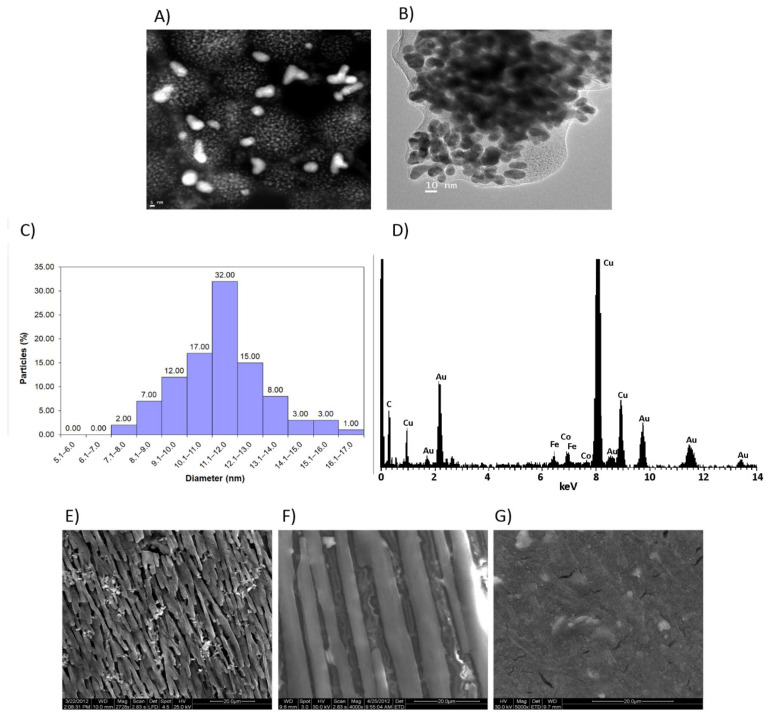
Micrographs of PANI-AuNPs nanocomposite obtained by (**A**) HAADF-STEM, and (**B**) TEM; (**C**) size distribution histogram; (**D**) EDS average spectrum. SEM micrographs of the different conducting polymer nanofibers and AuNPs nanocomposites obtained: (**E**) PANI-AuNPs, (**F**) PT-AuNPs and (**G**) PEDOT-AuNPs.

The morphology of the nanostructured conducting polymers was examined by SEM, TEM and HAADF-STEM techniques. Valuable information regarding the elements distribution on the surface of the PANI-AuNPs nanofibers was obtained by HAADF-STEM and EDS elemental analysis (see Figure 2). As examples, several TEM micrographs (Figure 2A,B) show different images of the PANI-AuNPs optimized material. AuNPs appear more brilliant (Figure 2A) (because of the contrast) than the lightest constituents of the conducting polymer. Importantly, the arrangement of the AuNPs within and throughout the surface of the nanostructure is clearly evidenced. The AuNPs immersed in the PANI nanofibers possess an average dimension of 11.2 ± 1.3 nm (measured from different TEM micrographs and using more than 100 AuNPs), with 91% of the population showing a size in the range 8–14 nm, as seen in the histogram of Figure 2C. Figure 2D corresponds to an EDS spectrum analysis of the PANI-AuNPs nanocomposite. According to the information provided by this technique, the elemental composition of the PANI-AuNPs nanocomposite was estimated as: 83.61 ± 5.88% of carbon, 2.27 ± 1.55% of nitrogen, 6.32 ± 2.22% of oxygen and 8.51 ± 5.06% of gold, all the values expressed in *w*/*w*. Thus, gold nanoparticles presence is also corroborated by these means.

On the other hand, the morphology of the different nanostructured conducting polymers was examined by SEM (see Figure 2E–G). The diameter of the PANI nanofibers can be controlled by changing the concentration of aniline, with lower concentrations producing fibers with thinner diameters, approximately 100 nm (as in the optimized sonosynthesis process). As commented previously, longer fibers will be more useful than shorter ones for electrical applications. Since the longer ones have less contact points than the shorter ones in the same unit distance, electrical conductivity with the longer nanofibers will be higher than that with the shorter nanofibers [39]. In our case, the diameter of the PANI nanofibers (approximately 100 nm) is lower than that obtained by other synthesis methods [46,47,48]. Particularly, for developing sensors and biosensors applications, polyaniline nanostructures with diameters lower than 200 nm are desired, and usually very complex dopants with bulky side groups are needed, such as sulfonated naphthalene derivatives [49], fullerenes [50] or dendrimers [51]. In the case of PT-AuNPs nanocomposites synthesized by the ultrasound-assisted method described in this paper, the diameter of the nanofiber was considerably higher, far from the desired 100 nm or less. Finally, the sonochemical synthesis of PEDOT-AuNPs generates even a less fibrillar structure, since monomer concentration used was lower and, subsequently, the monomer/KAuCl_4_ ratio was lower as well. Nonetheless, it should be noticed that both syntheses were successfully achieved in similar conditions, both providing satisfactory results. Because of these results, the PANI-AuNPs nanocomposite seems to offer better characteristics to confer good electrical conductivity for electrochemical application purposes, since it presents lower average diameter (ca. 100 nm) than those reported in the literature. That is why PANI-AuNPs nanocomposite is used in further studies.

**Figure 3:**
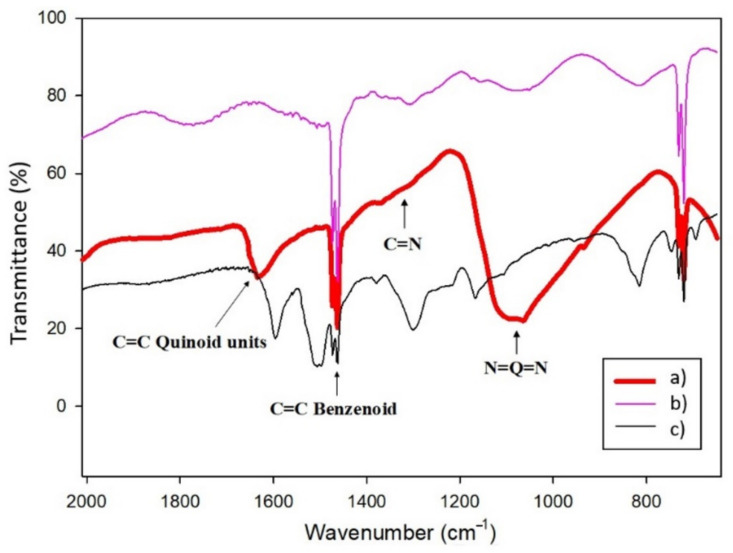
Infrared spectra of PANI-AuNPs synthesized using (a) 0.05 M LiClO_4_ as dopant and (b) without dopant, compared with (c) commercial polyaniline. Q = quinoid units.

Furthermore, structural characterization of the PANI-AuNPs nanocomposite was carried out by infrared spectroscopy, recording the main stretching bands in the range of 600–2000 cm^−1^ [30,52]. Figure 3 shows the FTIR spectra recorded with the PANI-AuNPs obtained using LiClO_4_ as dopant, without dopant and the commercial PANI. A characteristic peak of polyaniline emeraldine salt, located at 1630 cm^−1^, can be observed in the spectrum recorded with the composite synthesized with dopant, which can be ascribed to the C=C stretching of the quinoid rings. No peak can be noticed in the spectrum recorded with the one without dopant, which indicates the role of the dopant in the formation of conducting polyaniline. Furthermore, the peak located at 1140 cm^−1^ is related to the doped structure; this means that PANI-AuNPs nanostructure obtained by using sonochemical synthesis was more protonated than that resulting from the conventional polymerization, with a higher doping level leading to higher conductivity [53]. In the same way, this peak is not clearly distinguished in the spectrum corresponding to the polymer without dopant, confirming the key role of the dopant previously discussed.

The rest of the signals displayed with the PANI-AuNPs spectrum can be ascribed to the presence of polyaniline: the peak located at 1460 cm^−1^ can be assigned to the C-C stretching mode of the benzenoid rings. The small signal appreciated at about 1300 cm^−1^ may be ascribed to C-N stretching. All these assignments are in consonance with those reported in the literature for other Au/PANI composites [14,54,55].

After this structural characterization, it can be concluded that under the optimal conditions, it is possible to obtain bulk quantities of doped nCPs-AuNPs nanocomposites, without the need of conventional templates and following a fast, green, simple and one-pot process.

### 3.3. Electrochemical Characterization of the PANI-AuNPs Nanocomposite

#### 3.3.1. Electrode Capacity Assessment

As stated before, since PANI-AuNPs nanocomposite resulted in nanofibers with diameters lower than 100 nm, ideal for electrochemical (bio)sensing applications of this material, the electrochemical characterization studies were focused on this nanocomposite. For this purpose, certain volume from the aqueous solution of PANI-AuNPs was deposited on the surface of Sonogel-Carbon electrodes. Then, cyclic voltammograms were carried out in different media: 0.1 M phosphate-buffered solution (PBS) pH 7.2 and 0.1 M KCl. The electrochemical behavior of different PANI configurations was compared. These configurations were: (1) PANI-Au synthesized without the use of dopants, (2) PANI-Au-Li synthesized using 50 mM of LiClO_4_ as dopant, (3) PANI-Au-SDS synthesized using 5 mM of SDS as dopant, and (4) PANI-Au-Li+SDS synthesized using 50 mM of LiClO_4_ as dopant and 5 mM of SDS as stabilizer. This last configuration was tested seeking possible synergistic effects using both dopants.

**Figure 4:**
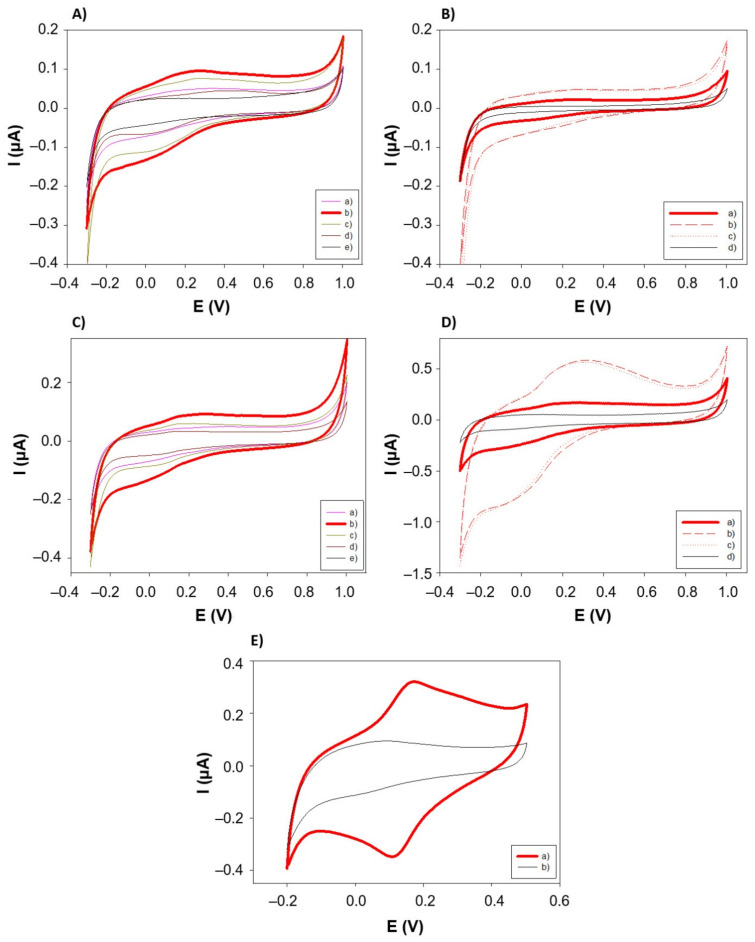
Cyclic voltammograms obtained for different PANI configurations recorded in different media and using different thicknesses of the nanocomposite material. (**A**) Different PANI configurations in 0.1 M of KCl, scan rate = 50 mV/s: (a) PANI-AuNPs obtained without using dopant, (b) PANI-AuNPs obtained using LiClO_4_ as dopant, (c) PANI-AuNPs obtained using SDS as dopant, (d) PANI-AuNPs obtained using LiClO_4_ as dopant and SDS as stabilizer, and (e) bare Sonogel-Carbon electrode. (**B**) Different thicknesses of PANI-AuNPs in 0.1 M of KCl, scan rate = 10 mV/s: (a) 2 µL, (b) 6 µL, (c) 10 µL, and (d) bare Sonogel-Carbon electrode. (**C**) Different PANI configurations in 0.1 M of PBS (pH 7.2), scan rate = 50 mV/s: (a) PANI-AuNPs obtained without using dopant, (b) PANI-AuNPs obtained using LiClO_4_ as dopant, (c) PANI-AuNPs obtained using SDS as dopant, (d) PANI-AuNPs obtained using LiClO_4_ as dopant and SDS as stabilizer, and (e) bare Sonogel-Carbon electrode. (**D**) Different thicknesses in 0.1 M of PBS (pH 7.2), scan rate = 100 mV/s: (a) 2 µL, (b) 6 µL, (c) 10 µL, and (d) bare Sonogel-Carbon electrode. (**E**) Electrochemical behavior in 0.1 M PBS (pH 7.2) of (a) PANI-AuNPs synthesized electrode using 0.05 M LIClO_4_ as dopant vs. (b) bare Sonogel-Carbon electrode. I(μA) = Current measured in microamperes and E(V) = potential measured in volts.

The electrochemical performances of these configurations in different media, with respect to the unmodified SNGC electrode, were evaluated (see Figure 4A,C). In all the electrode configurations containing PANI, the typical reversible redox behavior for this conducting polymer can be observed. Figure 4E specifically shows the signal comparison between the PANI-Au-Li configuration and the bare electrode. On the other hand, different thicknesses of these PANI configurations were also tested by depositing certain volumes onto the surface of the SNGC electrodes: 2, 6 and 10 µL. The CV signals obtained for each configuration at different thicknesses of the nanocomposite appear in Figure 4B,D. As can be seen, PANI-AuNPs nanocomposite-based SNGC electrodes show higher capacitive current than unmodified SNGCE, as expected.

The capacity corresponds to the non-faradaic current, i.e., the amount of charge that is not used to oxidize or reduce an electroactive specie. Experimental values of the observed capacity (*C_obs_*) at 100 mV/s and the double-layer capacity (*C_dl_*) for the different configurations of PANI-based SNGC electrodes were evaluated in absence of any analyte. The difference between them are as follows: on one hand the observed capacity is defined as (*C_obs_* = *i*/*ν*), where (*i*) is the average anodic and cathodic current density and (*ν*) is the scan rate, 100 mV/s; on the other hand, the double-layer capacity consists of the slope of the regression line obtained when the average values of the anodic and cathodic current densities are plotted at different scan rates versus the scan rate values. Both capacity values were calculated by taking the part of the voltammograms where non-faradaic currents are observed [56,57,58].

Values of *C_obs_* and *C_dl_* for different thicknesses of nanostructured conductive polymer deposited on the SNGC are shown in Table 1. In general, the lower capacity values enhance the electrochemical performance of the developed material. Besides, the presence of AuNPs allows the catalysis of electrochemical reactions that are not possible in the unmodified SNGC electrode. Independently of the electrolyte used, the capacity values for the electrode modified with the PANI-Au-Li nanocomposite (built using LiClO_4_ as dopant) is the highest one. This is due to, in this case, the dispersion generated during sonochemical synthesis leads to highly stable and less than 100 nm thickness nanofibers, with higher conductivity and, hence, higher capacitive values material. The other configurations show similar capacity values than those obtained for the bare SNGCE. When considering different volumes (and hence, thicknesses) of PANI-AuNPs nanocomposite deposited onto the SNGCE surface, the *C_obs_* values for 2, 6 and 10 μL measured in 0.1 M PBS were, respectively, 39.92, 112.17 and 104.88 μF/cm^2^. As can be observed, there is an increase in the capacity values with the volume deposited, which implies that higher amounts of polymer increase the capacitive resistance of the electrode. The lower value for the capacities corresponds to the unmodified SNGC electrode (see Table 1), as expected, since this parameter increases their values because of the presence of chemical species (modifiers) on the surface of the electrodes.

These values are in good agreement with those reported previously in the literature [58,59]. This result is also evident in Figure 4B,D; the higher the volume of nanocomposite deposited, the higher the distance between anodic and cathodic curves, resulting in an increasing capacity value. According to the results obtained, the best option for electrochemical measurements seems to be the deposition of 2 µL of PANI-AuNPs on the SNGC electrode, since this configuration offers closer capacity values to the bare SNGCE in PBS. Moreover, higher thicknesses of material could avoid efficient diffusion of electroactive species in electrochemical sensors, as well as of substrates in case of the nanocomposite is employed as immobilization matrix for building enzymatic amperometric biosensors.

#### 3.3.2. Mechanism and Impedance Studies

**Figure 5:**
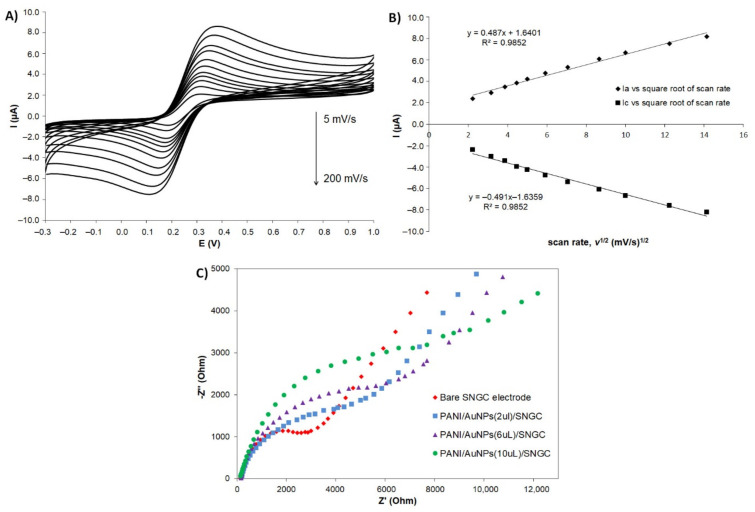
(**A**) Cyclic voltammograms corresponding to the Fe(III)/Fe(II) system at different scan rates: 5, 10, 15, 20, 25, 35, 50, 75, 100, 150 and 200 mV/s, in 5 mM of K_4_Fe(CN)_6_ in 500 mM of KNO_3_ solution. (**B**) Plots of anodic and cathodic peak currents versus ν^1/2^ (square root of the scan rate) for the SNGC electrode configuration of PANI-AuNPs using LiClO_4_ as dopant (2 µL of nanocomposite dispersion deposited) (**C**) Nyquist plots recorded at different thicknesses of LiClO_4_-doped PANI-AuNPs nanocomposite deposited on a bare SNGC electrode, and on 2 µL, 6 µL and 10 µL of PANI-AuNPs/SNGC electrode. Electrolyte: 5 mM of K_3_Fe(CN)_6_ and K_4_Fe(CN)_6_ in 500 mM of KNO_3_ solution. I(μA) = Current measured in microamperes; E(V) = potential measured in volts; and Z’ and Z’’ (Ohm) stands for the real part and the imaginary part of impedance (Z), respectively.

The electrochemical behavior of PANI-AuNPs configurations was also evaluated by CV in presence of a reversible redox system. The system used was composed of 5 mM of K_4_Fe(CN)_6_ in a 500 mM KNO_3_ solution. The PANI-Au-Li configuration exhibited greater intensity of anodic and cathodic current values when 2 µL of solution were deposited on the SNGC electrode surface. Figure 5A shows the cyclic voltammograms corresponding to the system Fe(III)/Fe(II) (5 mM), measured at a SNGC electrode with the PANI-Au-Li configuration, and at different scan rate values: 5, 10, 15, 20, 25, 35, 50, 75, 100, 150 and 200 mV/s. As expected, when increasing the scan rate value, both the anodic and cathodic intensity peak values also increase.

Moreover, there is a strong linear dependence between the current values and the square root of the scan rate, which implies a diffusion-controlled mechanism (Figure 5B). The *ΔE_p_* (=*E_a_* − *E_c_*) was 120 mV. Hence, it was higher than 59/*n* (mV) as expected for a quasi-reversible system [56,60]. From data plotted in Figure 5A, information about the area of the working electrode can be obtained. In general, and as it is well-known, the CV peak current *I_p_* (in A) of a diffusion-controlled reversible or quasi-reversible electrochemical reaction follows Randles–Sevcik equation, which at 25 °C is written in the form: *I_p_* = 2.69 × 10^5^·*n*^3/2^·*A*·*D*^1/2^·*c*·*ν*^1/2^, where *n* is the number of electrons, *A* is the electrode surface in cm^2^, *D* is the diffusion coefficient of the analyte (ferrocyanide) in cm^2^·s^−1^, *c* is the concentration in mol·cm^−3^ and *ν* is the scan rate in V·s^−1^. Hence, the effective area of the AuNPs-PANI/SNGC electrode is 2.13 × 10^−2^ cm^2^, whose value is double than the geometrical area, as stated in Section 2.4.

Similarly, assays were performed using electrochemical impedance spectroscopy (EIS) in presence of 5 mM of K_3_Fe(CN)_6_ and 5 mM of K_4_Fe(CN)_6_ in a 500 mM KNO_3_ solution, using the experimental conditions collected in Materials and Methods section. EIS is an effective method for probing the features of surface modified electrodes and can provide information on the impedance changes accompanying the stepwise electrode modification process [61]. In the Nyquist plot of impedance spectra, the semicircle portion at higher frequencies corresponds to the electron transfer limited process and the linear portion seen at the lower frequencies is due to the diffusion of electrons [59]. The electron transfer resistance at the electrode surface is equal to the semicircle diameter, which can be used to describe the interface characteristics of the electrode (see Figure 5C). From this plot, the corresponding values for the charge transfer resistance (*R_CT_*) were calculated as ca. 3.3, 5.9, 6.4 and 8.5 kΩ, for the bare SNGC, the PANI-AuNPs(2 µL)/SNGC, the PANI-AuNPs(6 µL)/SNGC and the PANI-AuNPs(10 µL)/SNGC electrodes, respectively. According to these results, the increasing order corresponding to the diameters of the semicircles, and hence, to the resistance is: bare < 2 < 6 < 10 µL of PANI-AuNPs. It is possible to conclude that 2 µL of PANI-AuNPs deposited provided the best conductivity. It also allowed better diffusion of the analyte, which is very important in the development of enzymatic amperometric biosensors [62].

#### 3.3.3. Benchmark Analyte Application

**Figure 6:**
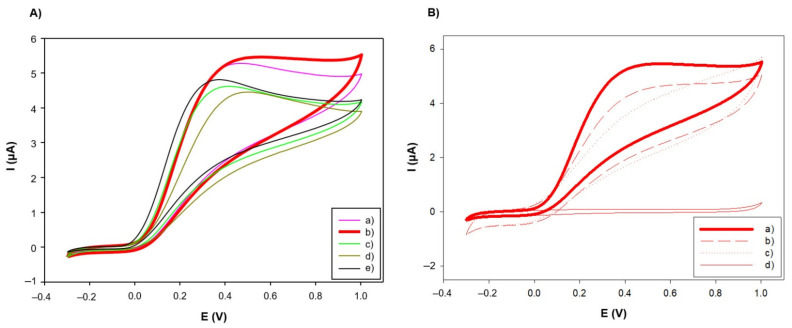
Cyclic voltammograms corresponding to 5 mM of ascorbic acid in 0.1 M PBS (pH 6.8). (**A**) Different PANI configurations: (a) PANI-AuNPs obtained without using dopant, (b) PANI-AuNPs obtained using LiClO_4_ as dopant, (c) PANI-AuNPs obtained using SDS as dopant, (d) PANI-AuNPs obtained using LiClO_4_ as dopant and SDS as stabilizer, and (e) bare Sonogel-Carbon electrode. (**B**) Different thicknesses of LiClO_4_-doped PANI-AuNPs nanocomposite deposited on a SNGC electrode: (a) 2 µL, (b) 6 µL, (c) 10 µL and (d) 2 µL PANI-AuNPs using LiClO_4_ as dopant without analyte. I(μA) = Current measured in microamperes and E(V) = potential measured in volts.

Finally, the behavior of the different PANI configurations in the detection of a benchmark electroactive species, such as ascorbic acid, was evaluated. Therefore, it is possible to evaluate the potential for using these materials as a base for the manufacture of amperometric electrochemical (bio)sensors. As seen in Figure 6A, when detecting 5 mM of ascorbic acid in PBS, the cyclic voltammograms show the characteristic irreversible behavior of this compound [60].

The current was higher in the case of the PANI-Au-Li configuration, which can be attributed to the increase in the effective area of the electrode when modified with the PANI-AuNPs nanocomposite. Concerning the thickness of the nanocomposite onto the electrode surface (Figure 6B), a better detection of ascorbic acid, with higher anodic current values, was observed when depositing 2 µL of the PANI-Au-Li configuration, which is in good agreement with the previous discussion: a higher amount of nanostructured conducting polymer deposited on the transducer surface increases the thickness of the diffusion outer barrier that the analyte must go through. Moreover, in the case of the lower thickness, the maximum of the peak is also slightly shifted to less positive potential values.

Summarizing, from the cyclic voltammetry and electrochemical impedance spectroscopy studies, it is possible to conclude that the way in which the PANI-AuNPs has been obtained (presence and kind of dopant) and the thickness of material deposited on the electrode surface influence its electrochemical behavior as in absence as in presence of electroactive species. In our case, it has been demonstrated that a 2 µL deposition of PANI-AuNPs nanocomposite synthesized by using LiClO_4_ as a dopant is the most appropriate configuration, since it exhibits considerably higher electroactivity compared with unmodified electrode and the other PANI configurations. This electroactivity can be attributed to the presence of a higher percentage of emeraldine form in the nanostructured conducting polymer, as concluded in the Structural Characterization section.

### 3.4. Application of GOX/PANI-AuNPs Biosensor for Glucose Detection

Based on the numerous studies reported in the literature, enzyme immobilization is one of the most important steps involved in the biosensor design [63]. The choice of the technique used for bonding the biological component (enzyme) to the transducer is essential, since the stability, longevity and sensitivity of the final device largely depend on the enzymatic layer configuration. Hence, in this paper, PANI-AuNPs nanocomposite has been employed as entrapping/immobilization matrix of glucose oxidase (GOX), used as benchmark enzyme, in order to study its electrochemical biosensing performance.

#### 3.4.1. Effect of Enzyme Loading

The GOX/PANI-AuNPs biosensor was built by using SNGC electrode as transducer and 2 µL of PANI-AuNPs nanocomposite as immobilization matrix. It is well-known that the amount of enzyme on the solid support can affect the sensitivity of the biosensor [64]. Therefore, in order to optimize the main characteristic of the device, the enzyme loading on the glucose biosensor was investigated by cyclic voltammetry. Different amounts of GOX were mixed with 10 µL of PANI-AuNPs solution and then, 2 µL of the mixture were deposited onto the SNGC electrodes. Thus, the devices had an enzymatic load of 7, 10 and 15 enzyme units/biosensor. Subsequently the response of the biosensors with the enzyme loading was assessed. The experiments were conducted by exposing each biosensor to 25 mL of 0.1 M phosphate-buffered solution pH 6.8, containing 0.5 mM (90 mg/L) of glucose.

**Figure 7:**
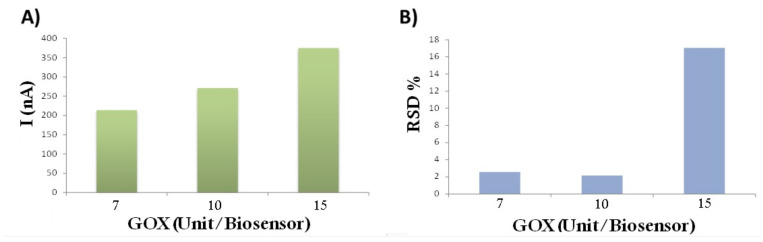
(**A**) Plot of current intensity versus the glucose oxidase enzyme loading (active units) per biosensor for a GOX/PANI-AuNPs(2 µL)/SNGC electrode (**B**) Relative standard deviation (n = 3). Experimental conditions: Cyclic voltammetry; 0.1 M phosphate-buffered solution pH 6.8 containing 0.5 mM of glucose.

The current intensity resulting from the addition of glucose was recorded (see Figure 7A,B), together with the relative standard deviation (RSD) of the current for 3 successive measurements for every case. As seen in the figure, the current intensity corresponding to the glucose catalysis by GOX increases with the enzyme loading (from 213 to 375 nA). However, RSD does this as well (from ca. 2% to 17%, for the 15 enzyme units/biosensor, which is considerably higher). Hence, in order to get a compromise between current intensity and RSD values, the most suitable amount of enzyme was 10 enzymes units/biosensor for the GOX/PANI-AuNPs electrochemical device.

#### 3.4.2. Electrochemical Assessment

**Figure 8:**
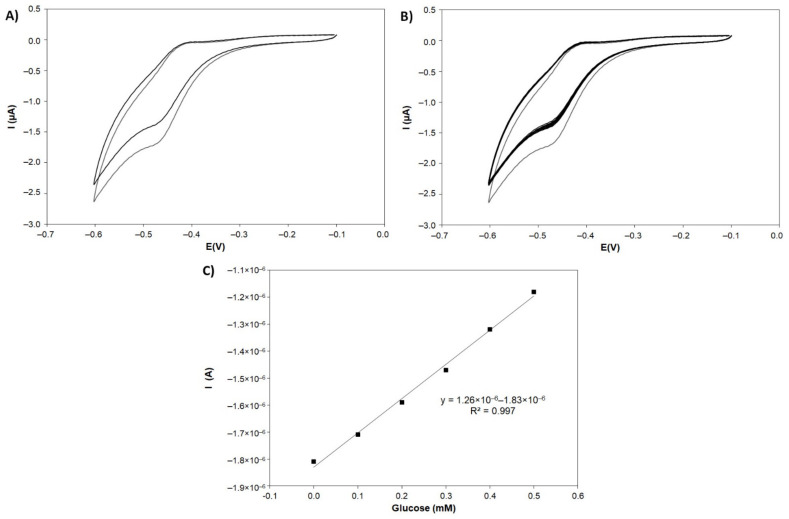
(**A**) Cyclic voltammograms of the GOX/PANI-AuNPs/SNGC biosensor in absence (grey) and in presence (black) of 0.5 mM of glucose in 0.1 M PBS (pH 6.8), scan rate 100 mV/s. (**B**) Cyclic voltammograms of the GOX/PANI-AuNPs/SNGC biosensor in absence (grey) and in presence (black) of 0.5 mM of glucose for ten replicates (repeatability study with an RSD value of 2.41%), in 0.1 M PBS (pH 6.8), scan rate 100 mV/s. (**C**) Calibration plot of glucose by using the GOX/PANI-AuNPs/SNGC biosensor in 0.1 M PBS (pH 6.8), scan rate 100 mV/s, for the concentration range from 0.1 to 0.5 mM. I(μA or A) = Current measured in microamperes or amperes and E(V) = potential measured in volts.

The electrochemical process developed by the GOX/PANI-AuNPs/SNGC biosensor can be observed in Figure 8A,B. In the absence of substrate (glucose), a redox wave is observed ca. −0.5 V vs. Ag/AgCl (3 M). In the presence of glucose, there is a repeatable decrease (RSD = 2.41% for n = 10) in the cathodic peak current (Figure 8B) due to the consumption of the redox active sites of the oxidized form of the enzyme in the reaction with the substrate. With increasing concentrations of glucose, a linear decrease in the cathodic peak current can be observed, and this fact can be used for the construction of a calibration plot (Figure 8C) from 0.1 mM (18 mg/L) to 0.5 mM (90 mg/L), interval where no oxygen interference was observed.

In addition, as the cathodic current decreases with the increment of glucose concentration and anodic peak current increases, it could be possible to assert that during the potential cycling, the active sites of the enzyme are reactivated.

#### 3.4.3. Calibration of Glucose

The GOX/PANI-AuNPs biosensor response vs. glucose was quantified by cyclic voltammetry in air-saturated PBS 0.1 M (pH 6.8). Five calibrations were performed for the same biosensor. The recorded current showed a good linear relationship in the range of 0.1–0.5 mM (18–90 mg/L). The sensitivity was 1.26 μA/mM (R^2^ = 0.997), showing a relative standard deviation (RSD) of 1.38%. The detection limit for the GOX/PANI-AuNPs biosensor was 0.056 mM (~10 mg/L), calculated as three times the standard deviation of the blank divided by the slope, as suggested by Miller and Miller [65].

A possible mechanism of the electrochemical reaction can be hypothesized from results. They indicate that competitive reactions through the direct electron transfer path towards oxygen reduction and glucose oxidation lead to a significantly decreased oxygen reduction current, allowing sensitive detection of glucose in a similar way as reported previously by Guo and Li [66], Li et al. [67] and Shan et al. [68] among many others.

The main quality analytical parameters of the GOX/PANI-AuNPs/SNGC biosensor were compared to those reported in the literature for similar glucose sensors (Table 2).

As seen in the table, the results obtained for the biosensor proposed in this paper are rather acceptable and are similar (or even better in some cases) to those reported previously in the literature. In fact, sensitivity is among the best values and the limit of detection is more than enough. Hence, it has been demonstrated that the PANI-AuNPs nanocomposite can be used for entrapping/immobilizing enzymes with great success, simplifying further the manufacturing of the biosensing device in some cases.

#### 3.4.4. Reproducibility and Stability Studies for Glucose Detection

Firstly, in absence of analyte, the variability of the current magnitude for five different biosensors was assessed, being the RSD equal to 2.41%. Secondly, the reproducibility of the biosensor was carried out for five different biosensors in a 0.5 mM (90 mg/L) standard glucose solution. The RSD was 4.46%, indicating that the fabrication process for the biosensor is reproducible.

The stability of the biosensor was also investigated. These characteristics are of vital importance when building biosensors. The stability tests were carried out at room temperature by cyclic voltammetry, measuring 3 times the responses of 0.5 mM (90 mg/L) of glucose every ten days. The biosensor was kept at 4 °C in buffer solution when not in use. The RSD was lower than 3 % after one month of storage: 0.61% the first day, 1.42% after ten days, 2.46% after twenty days and 2.93% after one month, indicating the good stability of the biosensor proposed. The good value of this analytical figure for the electronic device is due to the thinness and uniformity of the PANI-AuNPs film on the surface of the SNGC electrode. Thereby, the uniform nanostructure can significantly increase the effective surface of the electrode for loading enzymes and accelerating electron transfer kinetics. The film does not leak from the electrode and can stably adhere to the SNGC surface for a long time.

### 3.5. Real Wines Samples Analysis Employing the GOX/PANI-AuNPs/SNGC Biosensor

Finally, an application of the GOX/PANI-AuNPs/SNGC biosensor to real samples was accomplished. In this paper, we report the preliminary results concerning the determination of the glucose content in wine samples: a white and a red wine both collected from a local winemaker. The concentration of glucose was fixed at 0.5 mM (90 mg/L) in all wine samples. Each wine has a different glucose concentration, so dilution factors were properly applied with the aim of adding in the cell a wine aliquot that implies a final glucose concentration of approximately 0.5 mM (90 mg/L). Table 3 collects the results for the glucose content in the wine samples determined by a reference method (HPLC) and the GOX/PANI-AuNPs/SNGC biosensor.

As evidenced, the recovery percentages were very close to 100% and near the value taken as reference, demonstrating the high potential of the PANI-AuNPs nanocomposite when immobilizing GOX for the determination of glucose in real wine samples.

## 4. Future Perspectives

On one hand, sonocatalysis plays an important role in the synthesis of nanocomposites. Not only is this synthesis technique simple, fast and eco-friendly, but it also reduces costs significantly in terms of time and energy. In addition, from the scaling point of view high energy ultrasound based procedures are easy to implement, mainly for one-step and one-pot methodologies.

On the other hand, the applicability of this type of nanocomposites based on AuNPs and conducting polymers is enormous and susceptible to be included in both commercial and handmade electrochemical devices. For example, one possibility would be to incorporate these nanocomposite materials in electrode printing inks based on additive fabrication or 3D printing. The main aim would be to enhance the sensors performance and to facilitate the mass production, thus remarkably increasing the reproducibility of the process.

## 5. Conclusions

In this paper, a new synthesis procedure of conducting polymer nanofibers and gold nanoparticles nanocomposite is proposed. The green, one-pot and template-free method is based on the application of high energy ultrasound to an aqueous mixture of the monomer (aniline, thiophene or 3,4-ethylenedioxythiophene) and the metal salt (KAuCl_4_) in the presence of LiClO_4_ as dopant. The synthesis is very simple and fast, taking only a few minutes in comparison with other more complex systems and time-consuming procedures previously described. To the best of our knowledge, there is only one application of high energy ultrasound for the one-step synthesis of PANI-AuNPs nanocomposite, but without the good advantages as the present proposal reports. Under the optimal synthesis conditions, it is possible to obtain bulk quantities of doped PANI-AuNPs nanofibers at the more conductive oxidation state (emeraldine form), without the need of conventional templates, and with an average diameter appropriate for electroanalytical purposes (about 100 nm or lower). Furthermore, the application of PANI-AuNPs nanocomposites as entrapping/immobilization matrices for (bio)sensing purposes is successfully tested using GOX as a benchmark enzyme. PANI-AuNPs nanocomposite enhances biosensing performance due to the promotion of electron transfer between enzyme active sites and electrode surface and taking advantage of the combination of intrinsic features of conducting polymers and metal nanoparticles. The GOX/PANI-AuNPs/SNGC biosensor showed good repeatability, reproducibility, stability and electrochemical performance, since the quality analytical parameters obtained were comparable to the ones belonging to other similar biosensors reported previously in the literature, which in some cases, used Nafion or more complex architectures as an immobilization matrix. Moreover, this biosensor has been also applied to the determination of glucose in real samples of wine with excellent recovery percentages.

Finally, this study may offer important insights into the synthesis of nanostructured conducting polymers and also stimulates the exploration of the applications of these nanocomposites, especially in research fields such as (bio)sensors, catalysis and composite materials.

## Figures and Tables

**Table 1:** Experimental values of *C_obs_* at 100 mV/s and *C_dl_* for the different configurations of PANI-based SNGC electrodes: PANI-Au synthesized without the use of dopants, PANI-Au-Li synthesized using 50 mM of LiClO_4_ as dopant, PANI-Au-SDS synthesized using 5 mM of SDS as dopant, and PANI-Au-Li+SDS synthesized using 50 mM of LiClO_4_ as dopant and 5 mM of SDS as stabilizer.

PANI Configurations	0.1 M KCl		0.1 M PBS (pH 7.2)	
	*C_obs_* (µF/cm^2^)	*C_dl_* (µF/cm^2^)	*C_obs_* (µF/cm^2^)	*C_dl_* (µF/cm^2^)
PANI-Au	41.77	40.40	22.45	21.02
PANI-Au-Li	107.56	103.97	39.92	36.61
PANI-Au-SDS	57.91	55.04	26.46	25.09
PANI-Au-Li+SDS	33.52	32.48	15.46	14.71
Bare SNGC	44.11	43.18	14.78	14.21

**Table 2:** Comparison of analytical performance between GOX/PANI-AuNPs/SNGC electrode and other modified biosensors.

Glucose Biosensor ^1^	Linear Range (μM)	Detection Limit (μM)	Sensitivity (μA mM^−1^·cm^−2^)	Ref.
GOX/PANI-AuNPs/SNGC	100–500	56	30.36	This work
TNT/AuNPs|[Demim]Br|Nafion|GOX|GCE	10–1200	-	72.15	[69]
GOX/AuNPs-EGr/SPCE	50–1600	2.5	255	[70]
GOX/Au/GCE	60–130	0.32	-	[71]
CMCNFs-1000/GOX/GCE	100–2000	17.2	2.34	[72]
Nafion/GOX/CNF/AuE	150–2700	89	50	[73]
PMWCNT/GOX/GCE	200–5800	45	6.6	[74]
GOX-PEDOT/CFμE	500–15,000	-	8.5	[75]
GOX/cage-like-PbS/Nafion/GCE	50–1450	10	11.02	[68]
GOX/N-CNTE	Up to 6500	24	11	[76]

^1^ GOX: glucose oxidase; PANI: polyaniline; SNGC: Sonogel-Carbon; TNT: Titanate nanotubes; AuNPs; gold nanoparticles; [Demim]Br: brominated 1-decyl-3-methyl imidazole; GCE: glassy carbon electrode; EGr: electroactivated graphite; SPCE: screen-printed carbon electrode; Au: nanostructured gold film; CMCNFs-1000: coconut matrix carbon nanofiber aerogels calcinated at 1000 °C; CNF: carbon nanofibers; AuE: gold-plated quartz electrode; PMWCNT: pristine multiwalled carbon nanotubes; CFμE: carbon fiber microelectrode; N-CNTE: nitrogen-doped carbon nanotube electrode.

**Table 3:** Glucose content determined in two different wine samples (white and red wines) by using the GOX/PANI-AuNPs/SNGC biosensor in 0.1 M PBS (pH 6.8).

	Glucose Content (g/L)	
Wine sample	White Wine	Red Wine
Reference method (HPLC)	7.48	4.55
GOX/PANI-AuNPs/SNGC biosensor	7.51	4.49
RSD (%)	1.20	2.24
Recovery (%)	100.4	98.68

## Data Availability

Not applicable.
